# Short-term NAD^+^ supplementation prevents hearing loss in mouse models of Cockayne syndrome

**DOI:** 10.1038/s41514-019-0040-z

**Published:** 2020-01-07

**Authors:** Mustafa N. Okur, Beatrice Mao, Risako Kimura, Scott Haraczy, Tracy Fitzgerald, Kamren Edwards-Hollingsworth, Jane Tian, Wasif Osmani, Deborah L. Croteau, Matthew W. Kelley, Vilhelm A. Bohr

**Affiliations:** 1 Laboratory of Molecular Gerontology, National Institute on Aging, National Institutes of Health, Baltimore, MD 21224 USA; 2 Laboratory of Cochlear Development, National Institute on Deafness and Other Communication Disorders, Bethesda, MD 20892 USA; 3 Mouse Auditory Testing Core, National Institute on Deafness and Other Communication Disorders, Bethesda, MD 20892 USA; 4 Danish Center for Healthy Aging, University of Copenhagen, Blegdamsvej 3B, 2200 Copenhagen N, Denmark

**Keywords:** Neurodegenerative diseases, Genome

## Abstract

Age-related hearing loss (ARHL) is one of the most common disorders affecting elderly individuals. There is an urgent need for effective preventive measures for ARHL because none are currently available. Cockayne syndrome (CS) is a premature aging disease that presents with progressive hearing loss at a young age, but is otherwise similar to ARHL. There are two human genetic complementation groups of CS, A and B. While the clinical phenotypes in patients are similar, the proteins have very diverse functions, and insight into their convergence is of great interest. Here, we use mouse models for CS (*CSA*^−*/−*^ and *CSB*^*m/m*^) that recapitulate the hearing loss in human CS patients. We previously showed that NAD^+^, a key metabolite with various essential functions, is reduced in CS and associated with multiple CS phenotypes. In this study, we report that NAD^+^ levels are reduced in the cochlea of *CSB*^*m/m*^ mice and that short-term treatment (10 days) with the NAD^+^ precursor nicotinamide riboside (NR), prevents hearing loss, restores outer hair cell loss, and improves cochlear health in *CSB*^*m/m*^ mice. Similar, but more modest effects were observed in *CSA*^−*/−*^ mice. Remarkably, we observed a reduction in synaptic ribbon counts in the presynaptic zones of inner hair cells in both *CSA*^*−/−*^ and *CSB*^*m/m*^ mice, pointing to a converging mechanism for cochlear defects in CS. Ribbon synapses facilitate rapid and sustained synaptic transmission over long periods of time. Ribeye, a core protein of synaptic ribbons, possesses an NAD(H) binding pocket which regulates its activity. Intriguingly, NAD^+^ supplementation rescues reduced synaptic ribbon formation in both *CSA*^*−/−*^ and *CSB*^*m/m*^ mutant cochleae. These findings provide valuable insight into the mechanism of CS- and ARHL-associated hearing loss, and suggest a possible intervention.

## Introduction

Hearing loss is one of the most prominent age-associated conditions. Its prevalence almost doubles every decade starting from adult life and affects up to 80% of individuals over the age of 85.^1^ There are two major types of hearing loss: conductive and sensorineural. Any damage or obstruction that prevents sound from being conducted into the inner ear is considered conductive hearing loss. Sensorineural hearing loss derives from deficits in sensory cells or auditory nerves of the inner ear. A common type of sensorineural hearing loss is age-related hearing loss (ARHL), which occurs progressively in individuals as they age.^2^ Despite its high prevalence and cost, there are no interventions that prevent ARHL.

Cockayne syndrome (CS) is a premature aging disorder with prominent sensorineural hearing loss, similar to ARHL.^3,4^ CS is primarily caused by mutations in CSA and CSB proteins that participate in various biological processes including DNA repair, transcription, and mitochondrial functions.^5^ Hearing loss is a cardinal clinical symptom of CS, affecting up to 80% of CS patients by age 10.^4^ Hearing loss in CS resembles ARHL as both are bilateral, sensorineural, and progressive. Mouse models of CS (*CSA*^*−/−*^ and *CSB*^*m/m*^) recapitulate the progressive hearing loss and manifest sensory hair cell degeneration seen in the patients.^3,4,6^ Notably, hearing loss progression is slower in *CSA*^−*/−*^ than in *CSB*^*m/m*^ mice, reflecting the situation in CS patients.^4,6^

Our lab recently reported that the reduced abundance of nicotinamide dinucleotide (NAD^+^) in CS cells (patient-derived fibroblasts) and mice are associated with key CS phenotypes.^7^ NAD^+^ is a critical cofactor for several enzymes involved in mitochondrial biogenesis, mitophagy, and energy metabolism.^8^ It is likely that persistent DNA damage, observed in CS, constitutively activates poly-ADP ribose polymerase 1 (Parp1), which in turn depletes the cellular pool of NAD^+^.^7^ NAD^+^ declines with age and treatment with exogenous NAD^+^ improves mitochondrial function and life span in mice.^9–12^ Here, we investigate the relationship between NAD^+^ levels and hearing loss in CS mice. CS mice had lower levels of NAD^+^ so they were dosed with nicotinamide riboside (NR), an NAD^+^ precursor. NR has been shown to elevate cellular NAD^+^ levels in the cochlea and reduce neurite degeneration in auditory cells in mice following noise-induced damage.^13^

Hearing-related outcome measures used in this study included auditory brainstem response (ABR) and distortion product otoacoustic emission (DPAOE). ABR measures the fluctuation in voltage reflecting a neuronal response to sound, and DPOAE quantifies the electromotility of outer hair cells in the cochlea. The cochlea is the auditory portion of the inner ear and is composed of several cell types including inner and outer hair cells.^14^ Inner hair cells transduce sound vibration into electrical activity to be relayed into auditory nerve cells, while outer hair cells mechanically amplify low-level sound.

We find that NAD^+^ levels were reduced in the cochlea of *CSB*^*m/m*^ mice and that a brief intervention with NR (only 10 days) rescues progressive high-frequency hearing loss, improves outer hair cell survival, and normalizes DPAOEs in *CSB*^*m/m*^ mice. We observed similar but more modest effects on hearing loss in *CSA*^*−/*−^ mice following NAD^+^ intervention. Remarkably, we detected reduced numbers of synaptic ribbons in inner hair cells in both *CSA*^*−/−*^ and *CSB*^*m/m*^ mice, which were normalized after NAD^+^ supplementation. The assembly and function of synaptic ribbons in inner hair cells, which facilitate high vesicle turnover,^15^ are modulated by NAD^+^ and NADH.^16^ Therefore, these results provide insight into a converging mechanism underlying hearing loss in *CSA*^*−/*−^ and *CSB*^*m/m*^ mice, which may be similar to the mechanism underlying hearing loss in humans.

## Results

### Hearing in NR-treated CS mouse genotypes

**Fig. 1:**
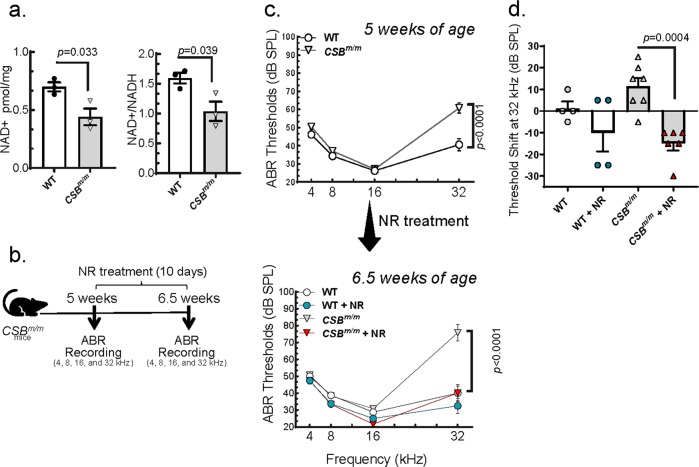
NAD^+^ supplementation prevents progressive hearing loss in *CSB*^*m/m*^ mice. **a** Total NAD^+^ levels per mg of the cochlea and relative NAD^+^/NADH levels were measured in the cochlea of *CSB*^*m/m*^ and WT (6-month-old) mice (*N* = 3). Two-tailed *t*-tests were used to determine significant difference. **b** Outline for NR treatment and ABR recordings in *CSB*^*m/m*^ mice. **c** ABR thresholds for *CSB*^*m/m*^ and WT mice following a 10-day treatment with NR (12 mM) delivered via drinking water. A total of 21 mice (*CSB*^*m/m*^ (*N* = 7); *CSB*^*m/m*^ + NR (*N* = 6); WT (*N* = 4); WT + NR (*N* = 4)) were tested by ABR at 5 weeks of age and again following the 10-day treatment (6.5 weeks of age). Multiple graphs are used to visualize error bars on each dataset. Two-way ANOVA with Tukey’s post-hoc test was used to determine significant difference. **d** Treatment with NR (12 mM) prevents the increased threshold shift observed in *CSB*^*m/m*^ mice between 5 weeks and 6.5 weeks at 32 kHz. Note: ABR data in **c** at 5 and 6.5 weeks of age were used to calculate the hearing shift. Two-way ANOVA with uncorrected Fisher's LSD post-hoc test was used to determine significant difference.

We previously reported that reduced NAD^+^ levels are associated with CS pathology.^7,17^ Given that hearing loss is a cardinal symptom of CS, we assayed NAD^+^ levels in the cochlea of *CSB*^*m/m*^ mice. We found that total NAD^+^ and relative NAD^+^/NADH levels were lower in the cochlea of the *CSB*^*m/m*^ mice compared to WT (Fig. 1a). In this study, CS mice were dosed with NR, an NAD^+^ precursor, to assess the effect of NAD^+^ supplementation on progressive hearing loss in CS mouse genotypes. We began treating mice with NR just after 5 weeks of age and assessed hearing capacity by measuring ABRs (see Methods for details) in response to sound at multiple frequencies (Fig. 1b). We observed that *CSB*^*m/m*^ mice at 6.5 weeks of age develop a progressive hearing impairment and have a hearing loss of more than 35 dB at 32 kHz compared to WT (Fig. 1c and Supplementary Fig. 1). Strikingly, only 10 days of exposure to NR (12 mM) significantly prevented the hearing loss in *CSB*^*m/m*^ mice and reduced the high-frequency hearing threshold from 77 to 40 dB (Fig. 1c, compare top and lower panels). We then compared the hearing threshold shift in individual mice in NR-treated and non-treated groups. We observed that non-treated *CSB*^*m/m*^ mice at 6.5 weeks of age, on average, lost 12 dB over 10 days and the treatment with NR not only prevented hearing loss, but improved hearing by an average of 15 dB (Fig. 1d).

**Fig. 2:**
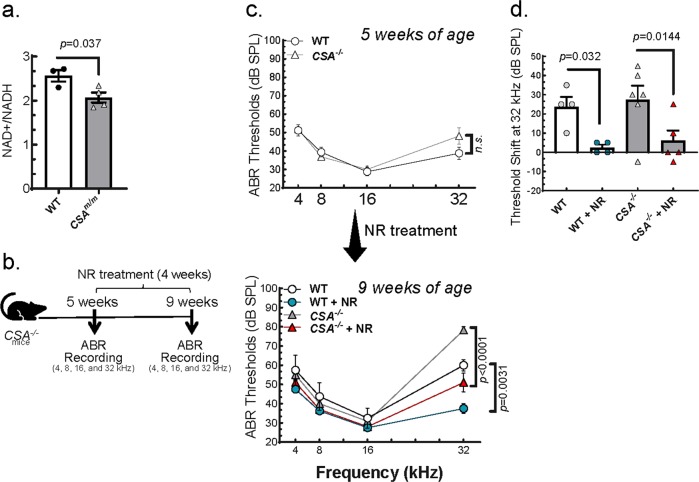
NAD^+^ supplementation prevents progressive hearing loss in *CSA*^*−/*−^ mice. **a** NAD^+^/NADH levels were measured in *CSA*^−*/−*^ (7-month-old) mice (*N* = 3). Two-tailed *t*-tests were used to determine significant difference. **b** Outline for NR treatment and ABR recordings in *CSA*^−*/−*^ mice. **c** ABR thresholds of *CSA*^−*/−*^ and WT mice following 4 weeks of NR treatment (24 mM) in their drinking water. Total of 19 mice (*CSA*^−*/−*^ (*N* = 6); *CSA*^−*/−*^ + NR (*N* = 5); WT (*N* = 4); WT + NR (*N* = 4)) were used to measure ABR at the age of 5 weeks (before NR treatment) and 9 weeks (after NR treatment). *CSA*^*−/−*^ mice do not show a significant threshold shift at any frequency at 5 weeks of age. However, at 9 weeks, *CSA*^−*/−*^ mice show an approximately 20-dB shift by comparison with WT. Multiple graphs are used to visualize error bars on each dataset. Two-way ANOVA with Tukey’s post-hoc test was used in to determine significant difference. **d** A significant threshold shift is present in *CSA*^−*/−*^ mice and WT at 32 kHz. Treatment with NR (24 mM) induced a significant rescue of the age-related threshold shift in *CSA*^−*/−*^ and WT mice. ABR data in **c** at 5 and 9 weeks of age were used to calculate the hearing shift. Two-way ANOVA with uncorrected Fisher's LSD post-hoc test was used to determine significant difference.

Like *CSB*^*m/m*^, *CSA*^*−/*−^ mice also manifested progressive hearing loss although less severe^6^ (Supplementary Fig. 2a). This less severe phenotype has also been reported in CSA patients.^4,6^ We found that NAD^+^/NADH levels were also lower in the cochlea of *CSA*^*−/*−^ mice (Fig. 2a) and tested the effect of NAD^+^ supplementation on the hearing defect in *CSA*^*−/*−^ mice (Fig. 2b). Initial treatment with 12 mM dose of NR did not attenuate the hearing loss in these mice (Supplementary Fig. 2b) and thus we increased the dose of NR (24 mM) over a 4-week period from 5 to 9 weeks of age. After exposure to 24 mM NR, the ABR threshold at 32 kHz for *CSA*^*−/−*^ decreased by ~25 dB (Fig. 2c, compare top and lower panels). During this 4-week period, non-treated *CSA*^*−/−*^ mice developed an average of 28 dB of hearing loss while the hearing threshold in NR-treated *CSA*^−*/*−^ mice was elevated only 6 dB, which is not considered as hearing loss (<10 dB) (Fig. 2d). These results collectively suggest that NAD^+^ supplementation strongly reduces hearing loss in both *CSA*^*−/*−^ and *CSB*^*m/m*^ mice.

### Outer hair cells in NR-treated CS mouse genotypes

**Fig. 3:**
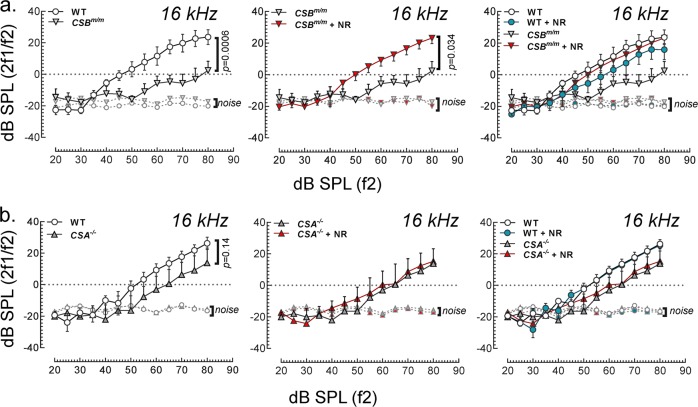
Short-term NR supplementation restores reduced DPOAE levels in *CSB*^*m/m*^ mice at 16 kHz. **a** NR intervention (12 mM of NR for 7 weeks) corrects reduced DPOAEs in *CSB*^*m/m*^ mice at 12 weeks of age at 16 kHz (*CSB*^*m/m*^ (*N* = 5); *CSB*^*m/m*^ + NR (*N* = 3); WT (*N* = 7); WT + NR (*N* = 6)). Multiple graphs are used to visualize error bars in each dataset. **b** DPOAE levels in *CSA*^*−/−*^ and WT at 9 weeks of age (*CSA*^*−/−*^ (*N* = 6); *CSA*^*−/−*^ + NR (*N* = 5); WT (*N* = 3); WT + NR (*N* = 4)) were recorded at 16 kHz following 4 weeks of NR treatment (24 mM) in their drinking water. Mean ± S.E. is shown in all graphs. Multiple graphs are used to visualize error bars on each dataset. The input in dB/output in dB functions in the range from 50 dB SPL to 60 dB SPL input were used to perform statistical analysis on groups, followed by a linear mixed-effect model on repeated-measures of DPOAEs in each group.

To gain more insight into the mechanisms by which NR reduces hearing loss in CS mouse genotypes, we assessed auditory capacity with DPOAE assay, an approach that provides information about cochlear integrity and outer hair cell function. The results show that *CSB*^*m/m*^ mice at 12 weeks of age have lower DPOAE signals than WT mice at 16 kHz (Fig. 3a, left panel), but not at 10 and 12 kHz (Supplementary Fig. 3a). This decrease at 16 kHz was normalized by exposure to NR (Fig. 3a, middle panel), suggesting that NAD^+^ supplementation improves outer hair cell function in *CSB*^*m/m*^ mice at 16 kHz. In the *CSA*^*−/−*^ mice at 9 weeks of age, the DPOAE response was similar to WT mice for all frequencies tested, suggesting that outer hair cell function in 9-week-old *CSA*^*−/−*^ mice are intact and functional (Fig. 3b and Supplementary Fig. 3b).

**Fig. 4:**
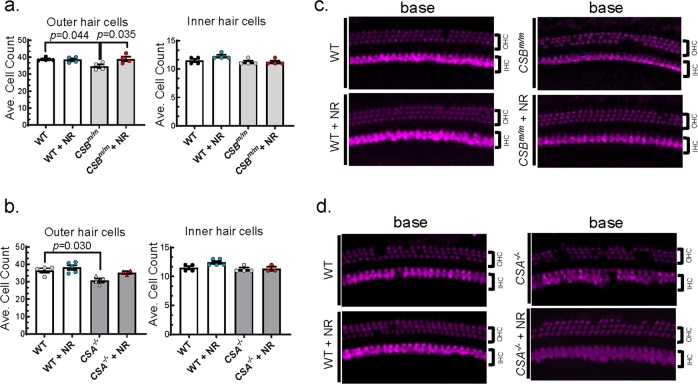
Outer hair cells are reduced in *CSA*^*−/−*^ and *CSB*^*m/m*^ mice, and short-term NR supplementation rescues outer hair cell loss in *CSB*^*m/m*^ mice. **a** Summary data for the average number of outer (left) and inner (right) hair cells per 100 μm in the basal region of the cochlea in WT or *CSB*^*m/m*^ mice with and without NR treatment (12 mM in drinking water for 10 days) at 6.5 weeks of age. The decreased number of outer hair cells observed in *CSB*^*m/m*^ mice is prevented by NR treatment. In contrast, no changes in the number of inner hair cells were observed between conditions. *N* = 4; mean ± S.E.; two-way ANOVA with Tukey’s post-hoc test was used to determine significant difference. **b** Representative images of outer and inner hair cells in the cochlear base region of *CSB*^*m/m*^ and WT mice following NR treatment as described in **a**. Myo7a staining is used to identify hair cells. **c** Outer hair cells were reduced in base region in *CSA*^*−/−*^ mice (*CSA*^*−/−*^ (*N* = 4); *CSA*^*−/−*^ + NR (*N* = 3); WT (*N* = 4); WT + NR (*N* = 5)). Mean ± S.E.; two-way ANOVA with Tukey’s post-hoc test was used to determine significant difference. **d** Representative images of base outer and inner hair cells of *CSA*^*−/−*^ and WT mice following NR treatment as described in **c**. Myo7a staining is used to identify hair cells.

Sensorineural hearing loss arises specifically from degenerative and functional changes in cochlear hair and auditory nerve cells, and loss of their synaptic connections.^18,19^ Next, we assessed the degenerative changes in cochlear hair cells in *CSB*^*m/m*^ and *CSA*^*−/−*^ mice via cochlear histology analysis. The cochlea is composed of base, middle, and apex regions; the base region senses high-frequency and the apex region senses low-frequency sounds. Since hearing loss in CS mice was restricted to high frequencies (Fig. 2), we focused on the basal region of the cochlea, although middle and apical regions were also analyzed (see Methods for details). Hair cell numbers in each area correlate with the hearing capacity for specific frequency ranges. To investigate the influence of NR on hair cell number and function, cochleae were isolated and stained with an antibody to Myo7a, a hair cell marker. There were significantly fewer outer hair cells in the basal cochlear region in *CSB*^*m/m*^ mice than in WT (Fig. 4a, b), while middle and apex regions were largely unaffected (Supplementary Fig. 4). This observation is consistent with the ABR data showing high-frequency hearing loss in *CSB*^*m/m*^ mice. Exposure to NR normalized outer hair cell numbers in the base cochlear region in *CSB*^*m/m*^ mice (Fig. 4a, b). The outer hair cell number was also reduced in the base cochlear region in *CSA*^*−/−*^ mice but did not increase significantly after treatment with NR (Fig. 4c, d). As was the case for *CSB*^*m/m*^, the numbers of outer hair cells in the apical and middle regions of the cochleae in *CSA*^*−/−*^ mice were not affected, which is consistent with our DPOAE and ABR results at lower frequencies (Supplementary Fig. 5). These results suggest that NAD^+^ supplementation restores outer hair cell number specifically in *CSB*^*m/m*^ mice.

### Ribbon synapses in NR-treated CS mouse genotypes

**Fig. 5:**
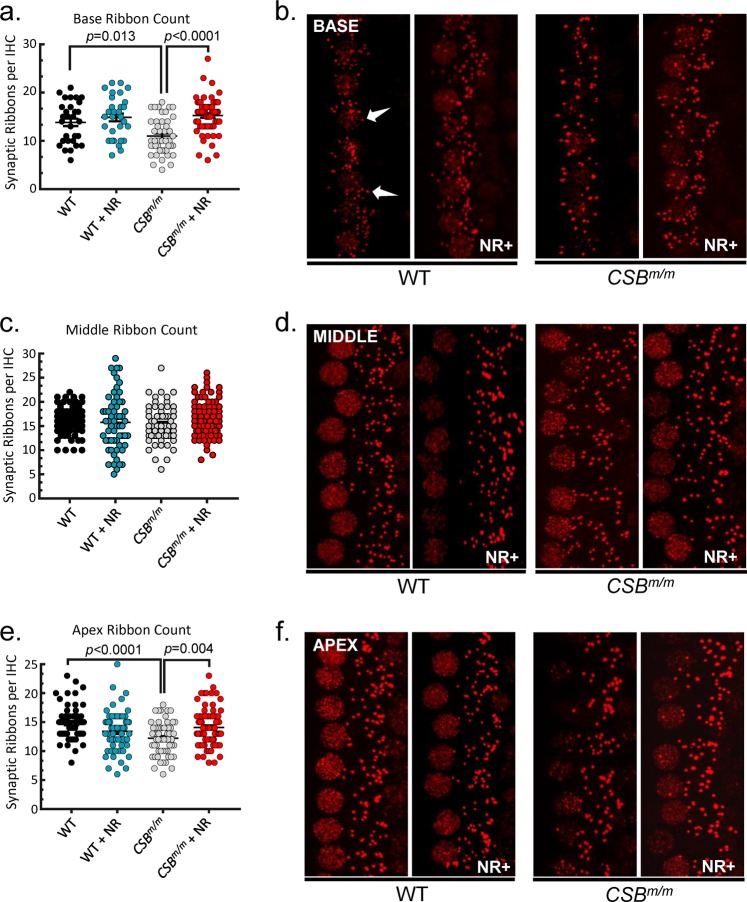
NR enhances synaptic ribbon count per inner hair cell in the base and apex regions in *CSB*^*m/m*^ mice cochlea. **a**, **c**, **e** The average synaptic ribbon count per inner hair cell in the cochlea base region (**a**), middle region (**c**), and apex region (**e**). The average number of ribbons is reduced in the base turn of the cochlea in *CSB*^*m/m*^ mice relative to WT at 6.5 weeks of age. However, this effect is prevented by NR treatment. A similar change is observed in the apical turn but not in the middle region of the cochlea. **b**, **d**, **f** Representative image of immunostaining for synaptic ribbons (red, anti-Ctbp2) of cochlear base segments (**b**), middle segments (**d**), and apex segments (**f**). A magnification of 40× was used for all images that are oriented such that the base of each hair cell is located on the right side of the image. Hair cell nuclei, which are also labeled with anti-Ctbp2, are located on the left side. Each individual puncta represents a single synaptic ribbon (arrows). Fifteen cells per region per mouse are used for quantification; 12 mM NR in drinking water for 10 days was used for intervention (*CSB*^*m/m*^ (*N* = 4); *CSB*^*m/m*^ + NR (*N* = 5); WT (*N* = 4); WT + NR (*N* = 4); mean ± S.E.; two-way ANOVA with Tukey’s post-hoc test was used to determine significant difference).

**Fig. 6:**
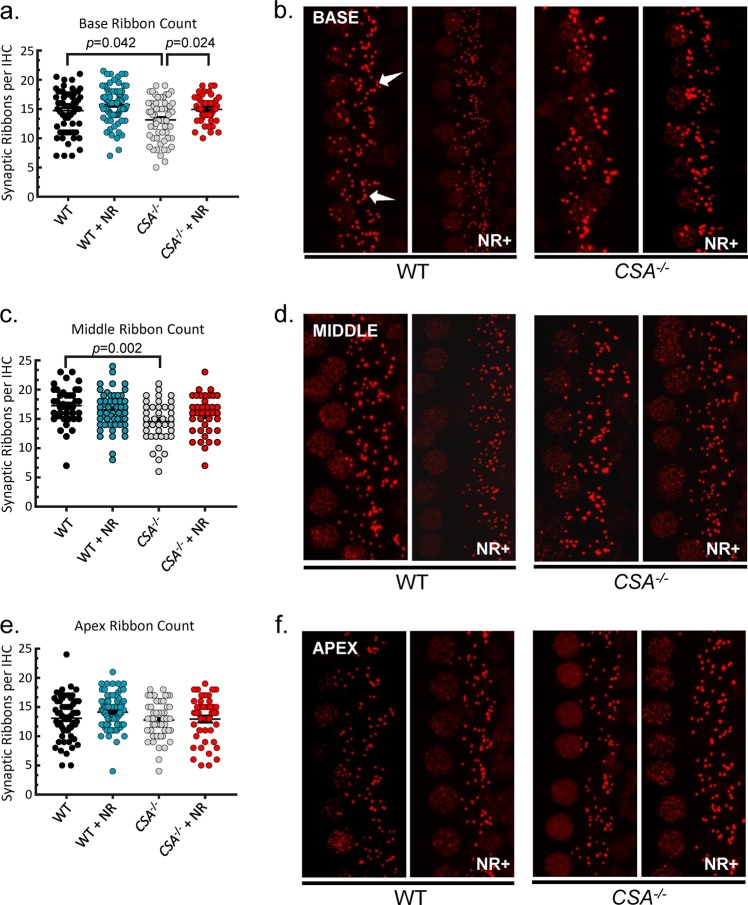
NR enhances synaptic ribbon count per inner hair cell in the base region in *CSA*^*−/−*^ mice cochlea. **a**, **c**, **e** The average synaptic ribbon count per inner hair cell in the cochlea base region (**a**), middle region (**c**), and apex region (**e**). The average number of ribbons is reduced in the base turn of the cochlea in *CSA*^*−/−*^ mice relative to WT at 9 weeks of age. However, this effect is prevented by NR treatment. **b**, **d**, **f** Representative image of immunostaining for synaptic ribbons (red, anti-Ctbp2) of cochlear base segments (**b**), middle segments (**d**), and apex segments (**f**). A magnification of 40× was used for all images that are oriented such that the base of each hair cell is located on the right side of the image. Hair cell nuclei, which are also labeled with anti-Ctbp2, are located on the left side. Each individual puncta represents a single synaptic ribbon (arrows). Fifteen cells per region per mouse are used for quantification; 24 mM NR in drinking water for 4 weeks was used for intervention (*CSA*^*−/−*^ (*N* = 4); *CSA*^*−/−*^ + NR (*N* = 4); WT (*N* = 4); WT + NR (*N* = 5); mean ± S.E.; two-way ANOVA with Tukey’s post-hoc test was used to determine significant difference).

The number of inner hair cells in all regions of the cochlea was similar in WT, *CSA*^*−/−*^, and *CSB*^*m/m*^ mice (Fig. 4a, c, right panels). We therefore next investigated the integrity of synaptic transmission between the inner hair cells and afferent neurons affecting the hearing loss in CS mouse genotypes. Inner hair cells form a specialized synapse, ribbon synapse, which provides rapid and sustained synaptic transmission.^15,20,21^ The function of ribbon synapses relies on the formation of unique electron-dense structures called synaptic ribbons, which are regarded as an accurate metric of inner hair cell afferent innervation.^15,20,22^ Interestingly, the major component of the synaptic ribbons is the protein Ribeye, which is important for the function and physical integrity of ribbon synapses, and it possesses a binding pocket for NAD(H).^23^ It has been shown that NAD(H) binding to Ribeye modulates the dimerization of Ribeye monomers to build the synaptic ribbon.^16,24^ Therefore, we tested whether NAD^+^ supplementation impacts ribbon formation in CS. We isolated cochleae and stained them with anti-Ctbp2 (Ribeye). Immunolabeling was quantified by counting ribbon-forming puncta in inner hair cells of WT, *CSA*^*−/−*^, and *CSB*^*m/m*^ mice. Compared to WT mice, *CSA*^*−/−*^ and *CSB*^*m/m*^ mice exhibited a significant reduction in ribbon counts of inner hair cells in the basal region of the cochlea, suggesting a defect in synaptic transmission in both genotypes of CS mouse (Figs. 5a, b and 6a, b). Remarkably, treatment with NR normalized ribbon counts in the same cochlear region of *CSA*^*−/−*^ and *CSB*^*m/m*^ mice (Figs. 5a, b and 6a, b). Synaptic ribbons were also reduced in the middle and apex cochlear regions in *CSA*^*−/−*^ and *CSB*^*m/m*^ mice, respectively (Figs. 5e, f and 6c, d). After intervention with NR, synaptic ribbons were normalized in the apex region in *CSB*^*m/m*^ but not in *CSA*^*−/−*^ mice. NR had no impact on the middle cochlear ribbons in either mouse strain (Figs. 5c, d and 6c, d). Although NR reduced hearing loss in WT mice (Fig. 2d), intervention with NR did not alter inner hair cell number/synaptic ribbon count in WT mice (Fig. 6a, b). Collectively, these results suggest that NR intervention restores impaired ribbon synapse formation, thereby improving synaptic transmission during the cochlear response to auditory stimuli to rescue high-frequency hearing loss in both genotypes of CS mouse.

## Discussion

Hearing loss is a major feature in CS patients. Here we sought to quantify hearing changes in CS mouse models and interrogate the potential utility of NAD^+^ supplementation. The results support the following conclusions: (1) Hearing loss progresses in *CSA*^*−/−*^ and *CSB*^m/m^ mice at high frequencies and short-term exposure to NR normalizes the ABR threshold at 32 kHz in *CSA*^*−/−*^ and *CSB*^*m/*m^ mice; (2) fewer outer hair cells are detected in the basal region of cochleae from *CSA*^*−/−*^ and *CSB*^*m/m*^ mice than in WT cochlea, and NR normalizes this phenotype in *CSB*^*m/m*^ mice; (3) the abundance of synaptic ribbons are lower in the base cochlear region from *CSA*^*−/−*^ and *CSB*^*m/m*^ mice than in control WT mice, and NR normalizes this phenotype in *CSA*^*−/−*^ and *CSB*^*m/m*^ mice. Although the hearing loss phenotype and its response to NR in WT and CS mice are complex, the data presented here support the idea that NAD^+^ depletion plays an important role in the etiology of age-dependent hearing loss in these mice.

CSA patients generally have a milder form of the disease than CSB patients.^5,6^ This is also recapitulated in the CSA mouse model which has less severe features than the CSB mouse.^6^ Importantly, both *CSA*^*−/−*^ and *CSB*^*m/m*^ mice show a dose-dependent reduction/normalization of hearing loss after exposure to NR (Supplementary Figs. 2b and 6). We next examined if there is a change in cochlear histology along with hearing loss prevention in NR-treated CS mice. To that end, we found that reduced DPOAEs in *CSB*^*m/m*^ mice at 16 kHz at 12 weeks of age were restored with NR (Fig. 3a). In line with the DPOAE results, we observed a reduction in the number of outer hair cells in the base turn of *CSB*^*m/m*^ cochlea at 6.5 weeks of age, and NR treatment rescued this abnormality (Fig. 4a, b). NAD^+^ supplementation corrects multiple aspects of mitochondrial abnormalities in the cerebellum of *CSB*^*m/m*^ mice.^7^ Perhaps, the same phenomenon occurs in *CSB*^*m/m*^ cochlea as well, enhancing outer hair cell survival via improving mitochondrial homeostasis. Interestingly, although there is a significant reduction of outer hair cells in the base region of *CSA*^*−/−*^ cochlea, this defect was not reflected in DPOAE levels in *CSA*^*−/−*^ mice (Figs. 3b and 4c, d). It is possible that loss of outer hair cells in *CSA*^*−/−*^ mice was compensated with increased function of nearby outer hair cells, thereby elevating DPOAEs. Nevertheless, unlike in *CSB*^*m/m*^ mice, NR treatment did not have a significant impact either on outer hair cells or on DPOAEs in *CSA*^*−/−*^ mice, suggesting that hearing benefits of NR treatment in this genotype does not include outer hair cell function or survival.

**Fig. 7:**
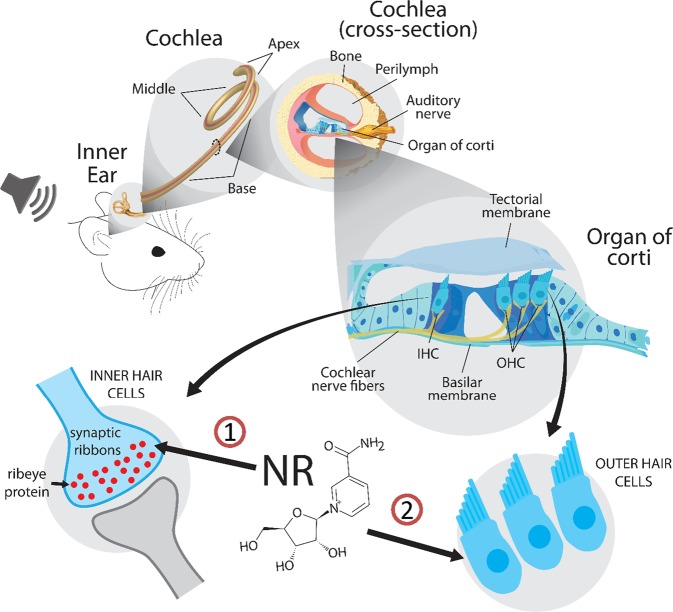
Figure demonstrating that NR intervention enhances synaptic ribbon formation in inner hair cells of *CSA*^*−/−*^ and *CSB*^*m/m*^ mice (arrow #1), and prevents outer hair cell loss in *CSB*^*m/m*^ mice (arrow #2). OHC stands for outer hair cells. IHC stands for inner hair cells.

Despite the dramatic hearing loss, the number of inner hair cells was largely unaffected in CS mice cochleae. Therefore, we assessed synaptic connectivity between inner hair cells and innervating afferent neurons. We observed that there were reduced synaptic ribbon (Ribeye) counts in the base turn of *CSA*^*−/−*^ and *CSB*^*m/m*^ cochlea compared to WT mice, suggesting that defects in the ribbon synapses might be a converging mechanism leading to hearing loss in CS. The reduction of synaptic ribbon count in inner hair cells was 11% in *CSA*^*−/−*^ mice by the age of 9 weeks and 21% in *CSB*^*m/m*^ mice by the age of 6.5 weeks at high frequencies (Figs. 5a and 6a). This amount of reduction in the synaptic ribbon counts occurs at approximately 32 and 80 weeks of age in mice with normal aging, respectively.^25^ Remarkably, NR intervention significantly corrected the reduced ribbon counts in both *CSA*^*−/−*^ and *CSB*^*m/m*^ cochlear inner hair cells (Figs. 5a, b and 6a, b). We conclude that depleted NAD^+^ levels due to deficiency of CS proteins disrupt synaptic ribbon assembly and function in inner hair cells. Thus, NAD^+^ supplementation may improve inner hair cell function via affecting ribbon synapses. This scenario is also consistent with the dramatic benefits of NR on hearing loss with short treatment duration (only 10 days) as replenishing NAD^+^ might exert its beneficial effect rapidly on synaptic ribbons and improve hearing. It is worth noting that synaptic ribbons are also present in cells of the retina.^26^ Interestingly, retinal dystrophy is another clinical feature of CS^4,27^, and NAD^+^ intervention protects against retinal degeneration in mice.^28^ Collectively, our results suggest that NAD^+^ supplementation via NR has distinct targets including synaptic ribbon formation in inner hair cells and the rescue of outer hair cell loss in CS cochlea (Fig. 7).

We previously reported that NAD^+^ supplementation can activate Sirt1 and rescue CS-related phenotypes.^7^ Sirt1 is a NAD^+^-dependent protein deacetylase and is known to have protective effects against aging-associated degeneration.^29^ Notably, Sirt1 expression also declines in the mouse cochlea during aging and its overexpression protects cochlear hair cells and delays early-onset ARHL. Given that Sirt1 activation is attenuated in *CSB*^*m/m*^ patient fibroblasts,^7^ the benefits of NAD^+^ supplementation on hearing in CS might also be mediated by Sirt1 activation. Indeed, Sirt1 activation by resveratrol treatment prevents outer hair cell loss and rescues the reduced synaptic ribbon counts in inner hair cells upon aging.^30,31^ Future studies exploring the CSB-Sirt1-NAD axis in the context of hearing loss are warranted as the role of Sirt1 on cochlear function is still a subject of debate.^32^

NR is generally recognized as safe for use in dietary supplements and well-tolerated up to 1000 g daily in humans for 6 weeks.^33^ Recent human clinical reports suggested potential benefits of NR intervention on improving redox homeostasis and exercise performance while reducing systolic blood pressure and arterial stiffness.^33,34^ Our study provides a rationale for exploring NR as a potential therapeutic approach for both accelerated and normal sensorineural hearing loss in humans. Hearing loss in CS resembles ARHL as both are sensorineural and progressive rather than congenital. Therefore, NAD^+^ supplementation might provide protective effects on hearing loss during aging as well. Indeed, the results presented here show that NR (24 mM dose) protects against hearing loss above 20 dB in WT mice (Fig. 2d). With the increasing prevalence of hearing loss in the aging population and the lack of therapeutic approaches for its treatment, the results of this study could ultimately, once successfully translated to the clinical arena, have a large public health impact. However, given that the cellular bioavailability of NAD^+^ declines upon aging,^11,12^ chronic NR supplementation may be necessary to achieve its full benefits. Notably, the hearing defect and beneficial results of the intervention are seen in both *CSA*^*−/−*^ and *CSB*^*m/m*^ mice. The proteins have very different functions that converge in our studies and thus may explain fundamental issues related to the CS disorder.

## Methods

### Animals

Mouse models of *CSA*^*−/−*^ (knockout mice),^35^ *CSB*^*m/m*^ (mice carrying a premature stop codon in exon 5 to mimic the K337 stop truncation mutation from CS patient (CS1AN)),^36^ and wild-type (WT) on a C57BL/6J background in the range of 5–12 weeks of age were used for the auditory and histology experiments. Six- or 7-month-old mice were used for quantifying NAD^+^ levels in the cochlea. The NR treatment started at the age of 5 weeks for both CS mice models right after auditory assessment. All animal protocols were approved by the Animal Care and Use Committee of the Intramural Research Program of the National Institute on Aging, in accordance with the National Research Council’s Guide for the Care and Use of Laboratory Animals.

### NAD^+^ supplementation

Unless stated otherwise, *CSB*^*m/m*^ mice were given NR at 3.5 mg/ml (12 mM) for 10 days and *CSA*^*−/−*^ mice were given NR at 7 mg/ml (24 mM) for 4 weeks in their drinking water, while the non-treated control groups received only drinking water. The drinking water supply was replenished/changed twice per week. A total of 1000 mg of NR daily supplementation for 6 weeks at this dose is well-tolerated in humans and effectively increases blood cellular NAD^+^ concentrations.^33^ The average 6 ml daily water intake of C57BL/6J mouse at an early age corresponds to the average of 21 mg (0.51 mg/kg) or 42 mg (1.02 mg/kg) daily NR intake in *CSB*^*m/m*^ and *CSA*^*−/−*^ mice, respectively.^37^ Given the difference in weight/surface area between human and mice,^38^ the range of 0.51–1.02 mg/kg of NR in mice corresponds to the range of 2.48–4.96 mg/kg of NR in humans, which is under the tolerated levels for NR consumption and is higher than the concentration needed to increase whole-blood NAD^+^.^33,39^

### NAD^+^ quantification

Following dissection in cold PBS, cochleae were weighed and placed in NADH/NAD Extraction Buffer (Abcam, ab65348). Following homogenization with a micro pestle, an NAD/NADH Assay Kit (Abcam, ab65348) was used to quantify NAD^+^ and NADH levels. Two technical replicates were performed on *n* = 3 mice per condition; data were normalized to cochlear weight.

### Audiometry

The ABR assay is a quantitative assessment of the neurological response, measured as evoked potential, detected within 10 ms of an auditory stimulus. ABR assays were performed on mice anesthetized with ketamine (100 mg/kg) and xylazine (10 mg/kg) via an intraperitoneal (i.p.) injection, placed on a heating pad 7 cm from the sound source, an MF1 Multi-Field Magnetic Speaker, in a soundproof chamber. Needle electrodes were inserted sub-dermally (vertex–ventrolateral to pinna). Tone burst stimuli (5 ms duration with a 0.1-ms rise–fall time) were presented at variable volume (10–90 dB SPL) in 5 dB steps at 4, 8, 16, and 32 kHz. The outcome measure is the minimum volume threshold (in dB) that evokes a response at each frequency tested. For each frequency, the threshold was determined by an average of 512 responses. ABR threshold was determined by visual inspection of the averaged waveforms. The threshold was considered to be the lowest stimulus level at which at least one wave was considered to be present. The threshold was the lowest dB stimulus that evoked at least one response wave. ABR assays were performed using an RZ6 system (Tucker Davis Technologies) with Biosig software (Tucker Davis Technologies).

DPOAE quantifies the electromotility of outer hair cells in the cochlea. DPOAEs were measured by inserting an ear-plug containing a small microphone (ER-10B+) and two speakers (MF1 Multi-Field Magnetic Speaker), in the outer ear canal of each mouse. A series of auditory stimuli were delivered, each composed of two tones at equal decibel level but distinct frequencies, *f*1 and *f*2, where *f*2 > *f*1, *f*2/*f*1 = 1.2) at *f*0 = 10, 12, 16, and 32 kHz (*f*0 = (*f*1 × *f*2)1/2). Decibel level of both tones varied over the range 80 dB SPL to 10 dB SPL in 5-dB steps. The cubic distortion product at the frequency 2*f*1 − *f*2 was recorded as an average of 512 responses at each frequency tested. DPOAE measurements were made using the RZ6 system (Tucker Davis Technologies) with Biosig software.

### Whole-mount cochlea immunostaining

Entire otic capsules were dissected and decalcified in a 0.25 M EDTA solution that was changed daily for 5–7 days. Subsequently, cochleae were dissected and divided into the apex, middle, and basal turns. Following decalcification and dissection, apex, middle, and basal turns of cochlea were blocked for 1 h with gentle agitation at room temperature in PBS, 20% triton X-100 (1:200), 10% normal donkey serum, and donkey anti-mouse fab fragments (Jackson ImmunoResearch Laboratories Inc., catalog #715-007-003, 1:200) with agitation. The primary antibody was added and incubated overnight at 4 °C. Primary antibodies were Myo7a (rabbit, Proteus Biosciences Inc., catalog #25-6790, 1:2000), and Ctbp2 (mouse, BD Biosciences, catalog #612044). After washing, the secondary antibody was added, and samples were incubated for 1 h at room temperature. Donkey secondary antibodies conjugated to Alexa Fluor fluorophores (Fisher Scientific, 1:1000) were used with phalloidin-Atto 390 (Sigma Aldrich, catalog #50556, 1:200) to stain filamentous actin or DAPI (4',6-diamidino-2-phenylindole, dihydrochloride, catalog #D1306, Thermo Fisher Scientific, 1:10,000) to label nuclei. Samples were mounted on glass slides in Vectashield antifade mounting medium (Vector Labs, catalog# H-1000), then imaged on a Zeiss 710 confocal microscope.

The total cochlear length was measured using ZEISS ZEN Black 2.3 SP1 software. Apex, middle, and base midpoints were assigned as loci at 25%, 50%, and 75% of the total length of the cochlea, respectively. Hair cell counts were quantified in 100-μm segments of the total cochlea for all regions (manual counting). For segments with damaged or immeasurable tissue, the nearest 150 µm of intact tissue was used. For synaptic counts, the number of presynaptic terminals in 15 inner hair cells was determined.

### Statistical analysis

Two-way ANOVA with Tukey’s post-hoc test was used to determine significant differences across multiple samples with two groups unless indicated otherwise. Two-way ANOVA with uncorrected Fisher's LSD post-hoc test was used to determine the significant difference in the hearing threshold when a comparison between groups was not involved. One-way ANOVA with Tukey’s post-hoc test was used to determine significant differences across multiple samples in more than two groups. Two-tailed *t*-tests were used to compare single groups. Statistical analyses were performed with GraphPad Prism (GraphPad Software, Inc.). The area under the curve across the whole dB SPL (*f*2) above the noise levels and a linear mixed-effect model for repeated-measures of DPOAE with multiple comparisons at the range of 50–60 dB SPL (*f*2) were employed to determine significant analysis for DPOAE levels between groups and both methods provided the same results.

### Reporting summary

Further information on research design is available in the Nature Research Reporting Summary linked to this article.

## Supplementary information

Suppl Figs

reporting summary

## Data Availability

The datasets generated during and/or analyzed during the current study are available from the corresponding author on reasonable request.
