# Supplementary material for: Short-term NAD+ supplementation prevents hearing loss in mouse models of Cockayne syndrome
Source: NPJ Aging Mech Dis. 2020 Jan 7;6:1. doi: 10.1038/s41514-019-0040-z (PMC6946667; doi:10.1038/s41514-019-0040-z)
Supplement: Supplementary file 1 — Suppl Figs [file 41514_2019_40_MOESM1_ESM.pptx]

## Slide 1
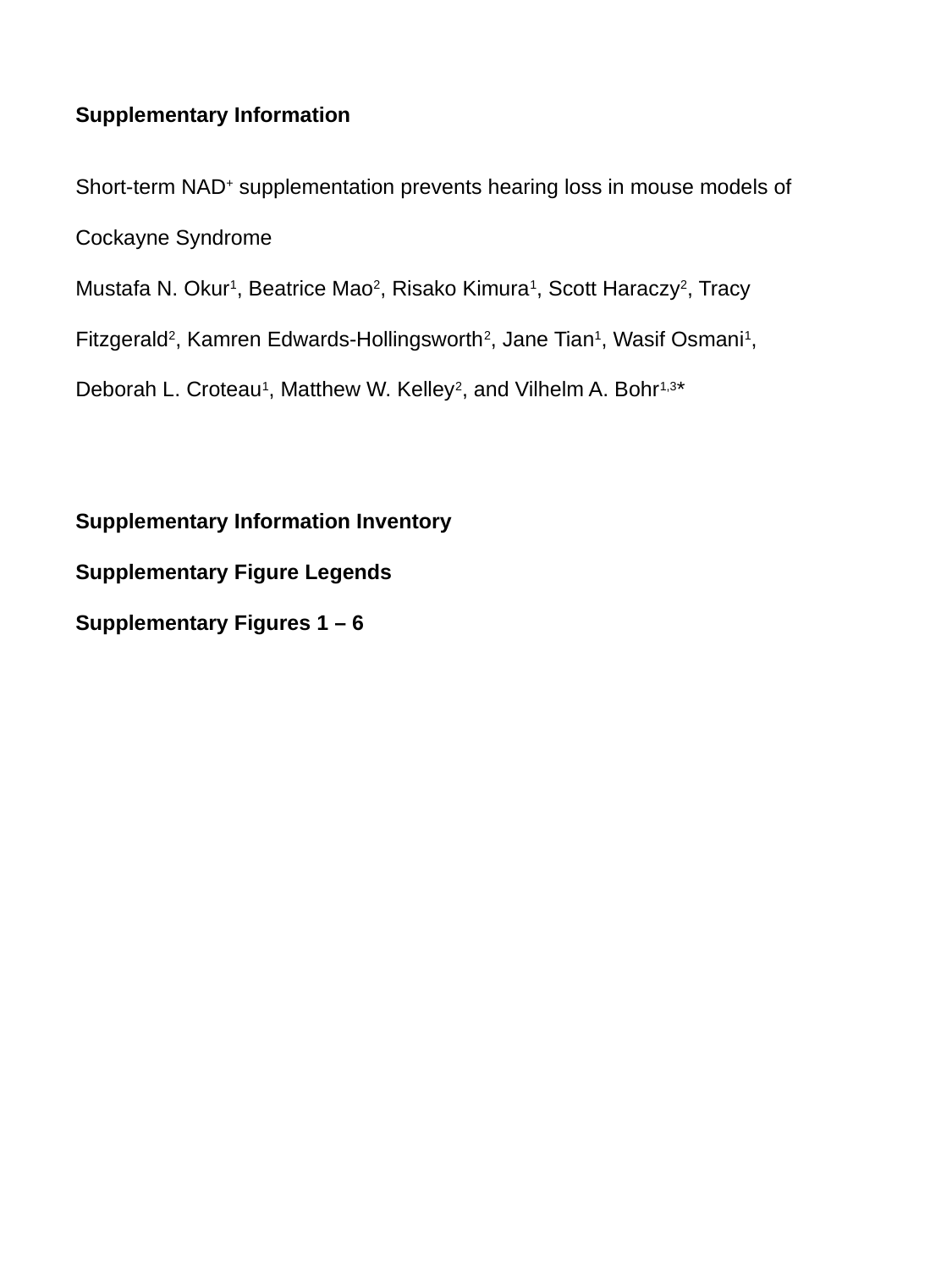

Supplementary Information
Short-term NAD+ supplementation prevents hearing loss in mouse models of Cockayne Syndrome
Mustafa N. Okur1, Beatrice Mao2, Risako Kimura1, Scott Haraczy2, Tracy Fitzgerald2, Kamren Edwards-Hollingsworth2, Jane Tian1, Wasif Osmani1, Deborah L. Croteau1, Matthew W. Kelley2, and Vilhelm A. Bohr1,3*
Supplementary Information Inventory
Supplementary Figure Legends
Supplementary Figures 1 – 6

## Slide 2
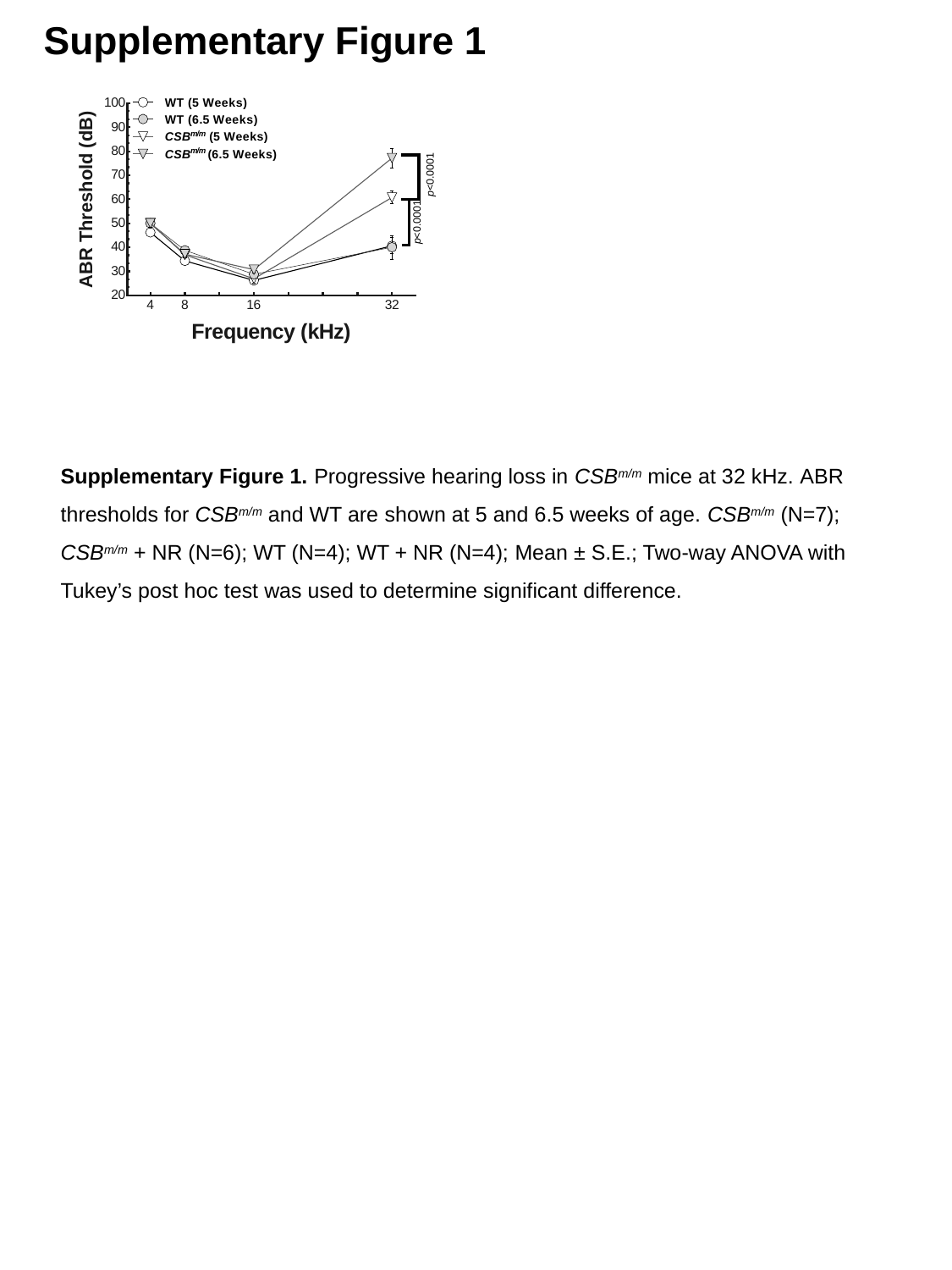

Supplementary Figure 1
p<0.0001
p<0.0001
Supplementary Figure 1. Progressive hearing loss in CSBm/m mice at 32 kHz. ABR thresholds for CSBm/m and WT are shown at 5 and 6.5 weeks of age. CSBm/m (N=7); CSBm/m + NR (N=6); WT (N=4); WT + NR (N=4); Mean ± S.E.; Two-way ANOVA with Tukey’s post hoc test was used to determine significant difference.

## Slide 3
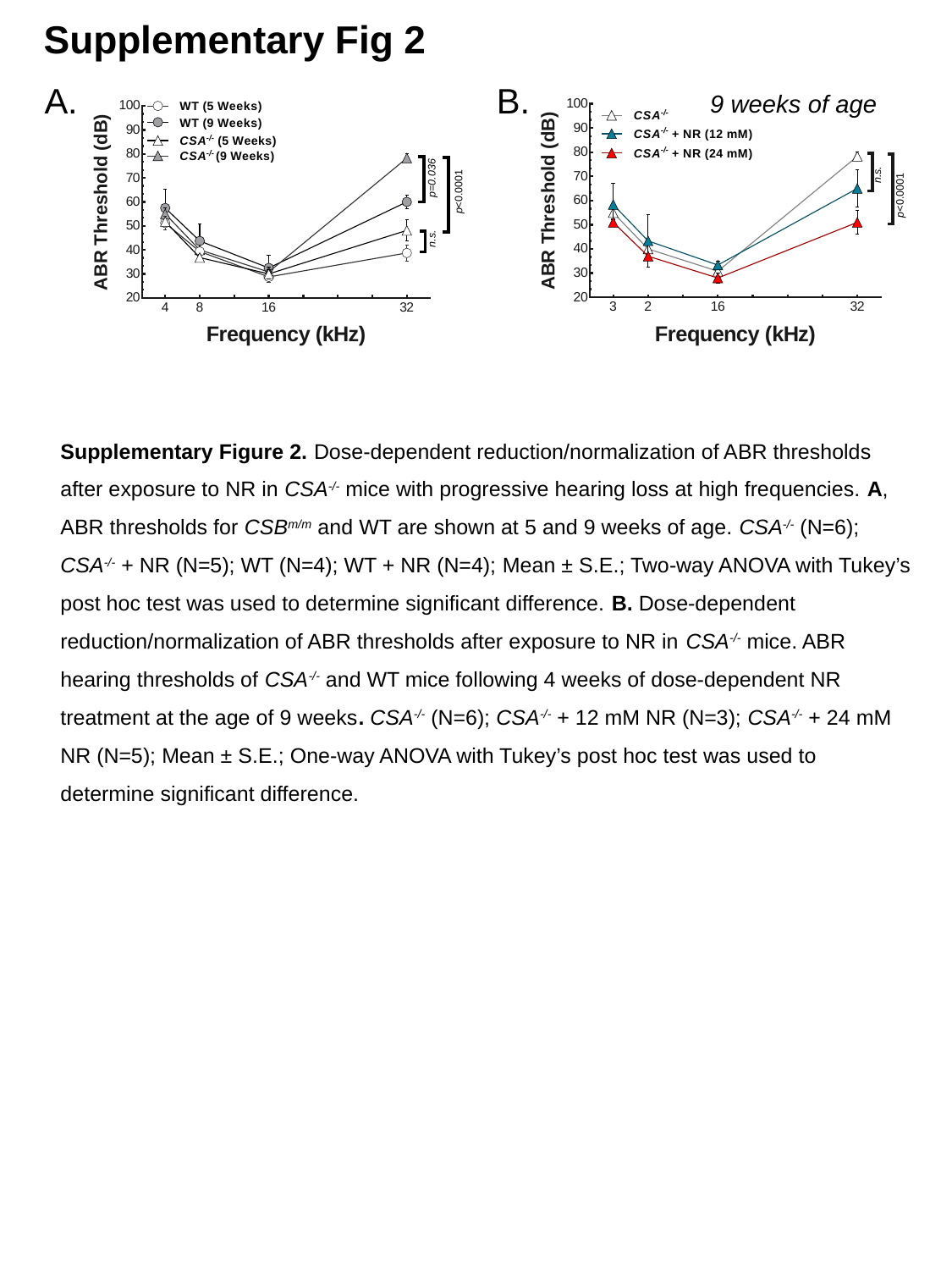

Supplementary Fig 2
9 weeks of age
n.s.
p<0.0001
A.
B.
p=0.036
p<0.0001
n.s.
Supplementary Figure 2. Dose-dependent reduction/normalization of ABR thresholds after exposure to NR in CSA-/- mice with progressive hearing loss at high frequencies. A, ABR thresholds for CSBm/m and WT are shown at 5 and 9 weeks of age. CSA-/- (N=6); CSA-/- + NR (N=5); WT (N=4); WT + NR (N=4); Mean ± S.E.; Two-way ANOVA with Tukey’s post hoc test was used to determine significant difference. B. Dose-dependent reduction/normalization of ABR thresholds after exposure to NR in CSA-/- mice. ABR hearing thresholds of CSA-/- and WT mice following 4 weeks of dose-dependent NR treatment at the age of 9 weeks. CSA-/- (N=6); CSA-/- + 12 mM NR (N=3); CSA-/- + 24 mM NR (N=5); Mean ± S.E.; One-way ANOVA with Tukey’s post hoc test was used to determine significant difference.

## Slide 4
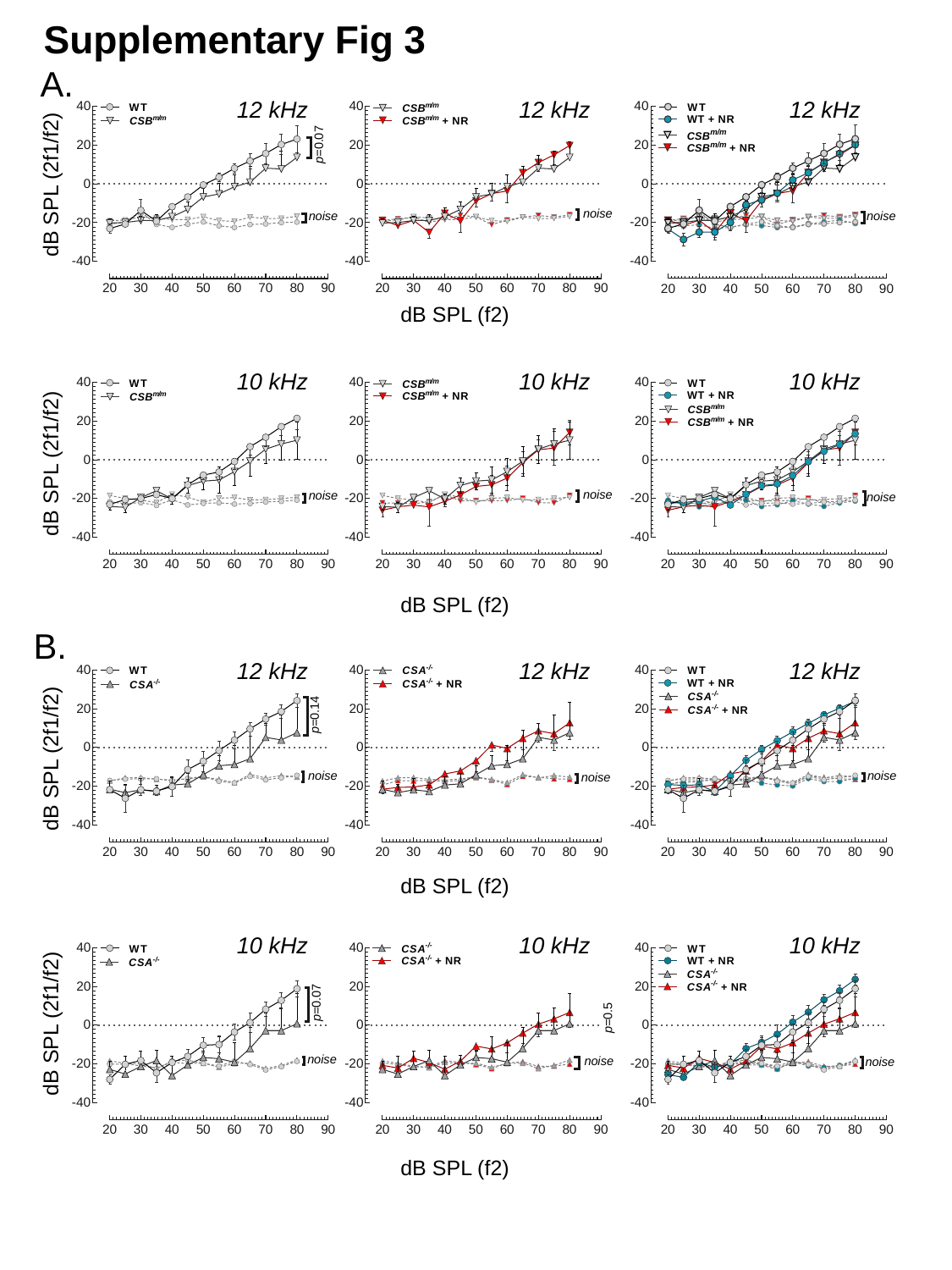

Supplementary Fig 3
A.
12 kHz
12 kHz
12 kHz
p=0.07
dB SPL (2f1/f2)
noise
noise
noise
dB SPL (f2)
10 kHz
10 kHz
10 kHz
dB SPL (2f1/f2)
noise
noise
noise
dB SPL (f2)
B.
12 kHz
12 kHz
12 kHz
p=0.14
dB SPL (2f1/f2)
noise
noise
noise
dB SPL (f2)
10 kHz
10 kHz
10 kHz
p=0.07
p=0.5
dB SPL (2f1/f2)
noise
noise
noise
dB SPL (f2)

## Slide 5
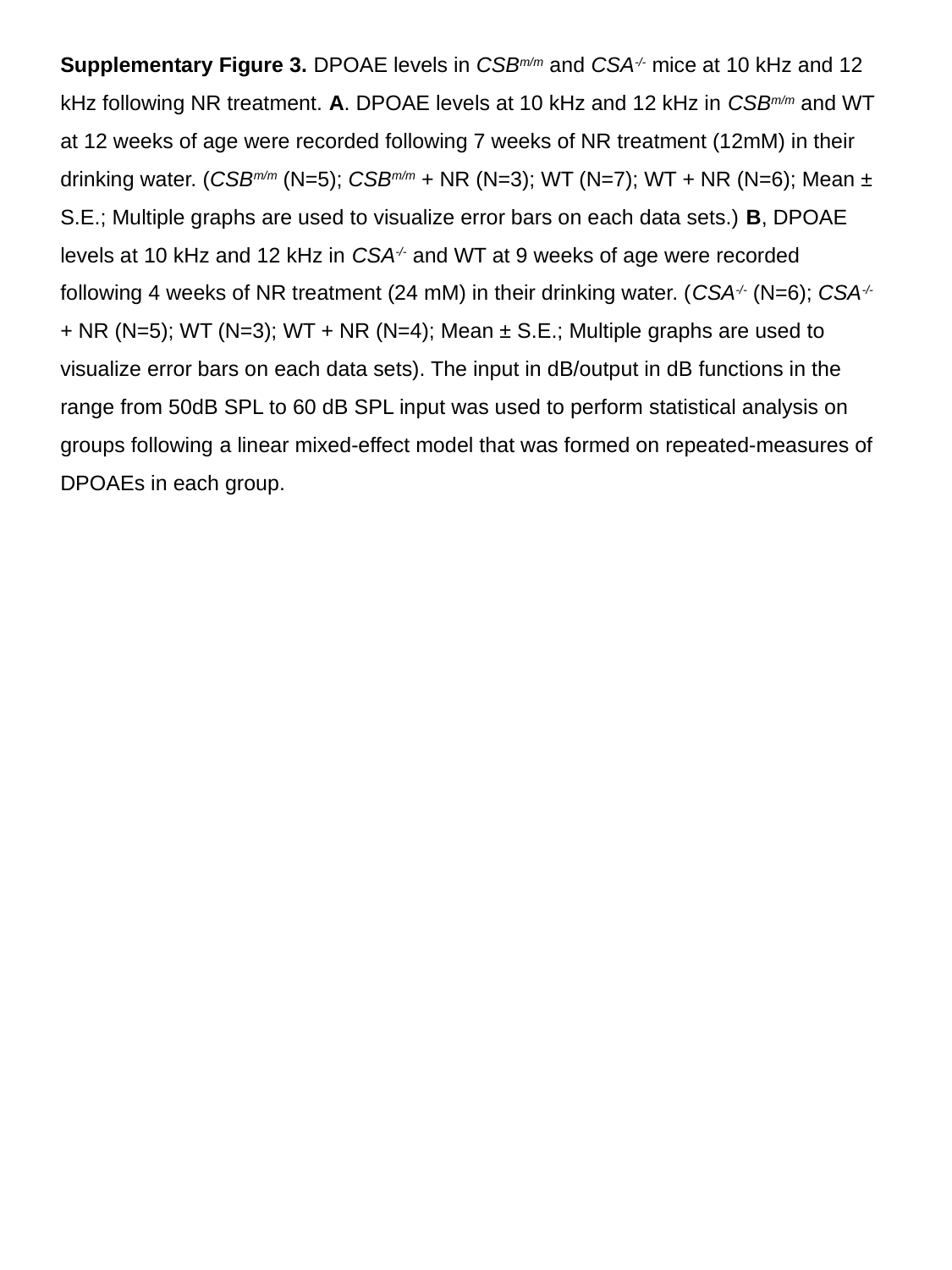

Supplementary Figure 3. DPOAE levels in CSBm/m and CSA-/- mice at 10 kHz and 12 kHz following NR treatment. A. DPOAE levels at 10 kHz and 12 kHz in CSBm/m and WT at 12 weeks of age were recorded following 7 weeks of NR treatment (12mM) in their drinking water. (CSBm/m (N=5); CSBm/m + NR (N=3); WT (N=7); WT + NR (N=6); Mean ± S.E.; Multiple graphs are used to visualize error bars on each data sets.) B, DPOAE levels at 10 kHz and 12 kHz in CSA-/- and WT at 9 weeks of age were recorded following 4 weeks of NR treatment (24 mM) in their drinking water. (CSA-/- (N=6); CSA-/- + NR (N=5); WT (N=3); WT + NR (N=4); Mean ± S.E.; Multiple graphs are used to visualize error bars on each data sets). The input in dB/output in dB functions in the range from 50dB SPL to 60 dB SPL input was used to perform statistical analysis on groups following a linear mixed-effect model that was formed on repeated-measures of DPOAEs in each group.

## Slide 6
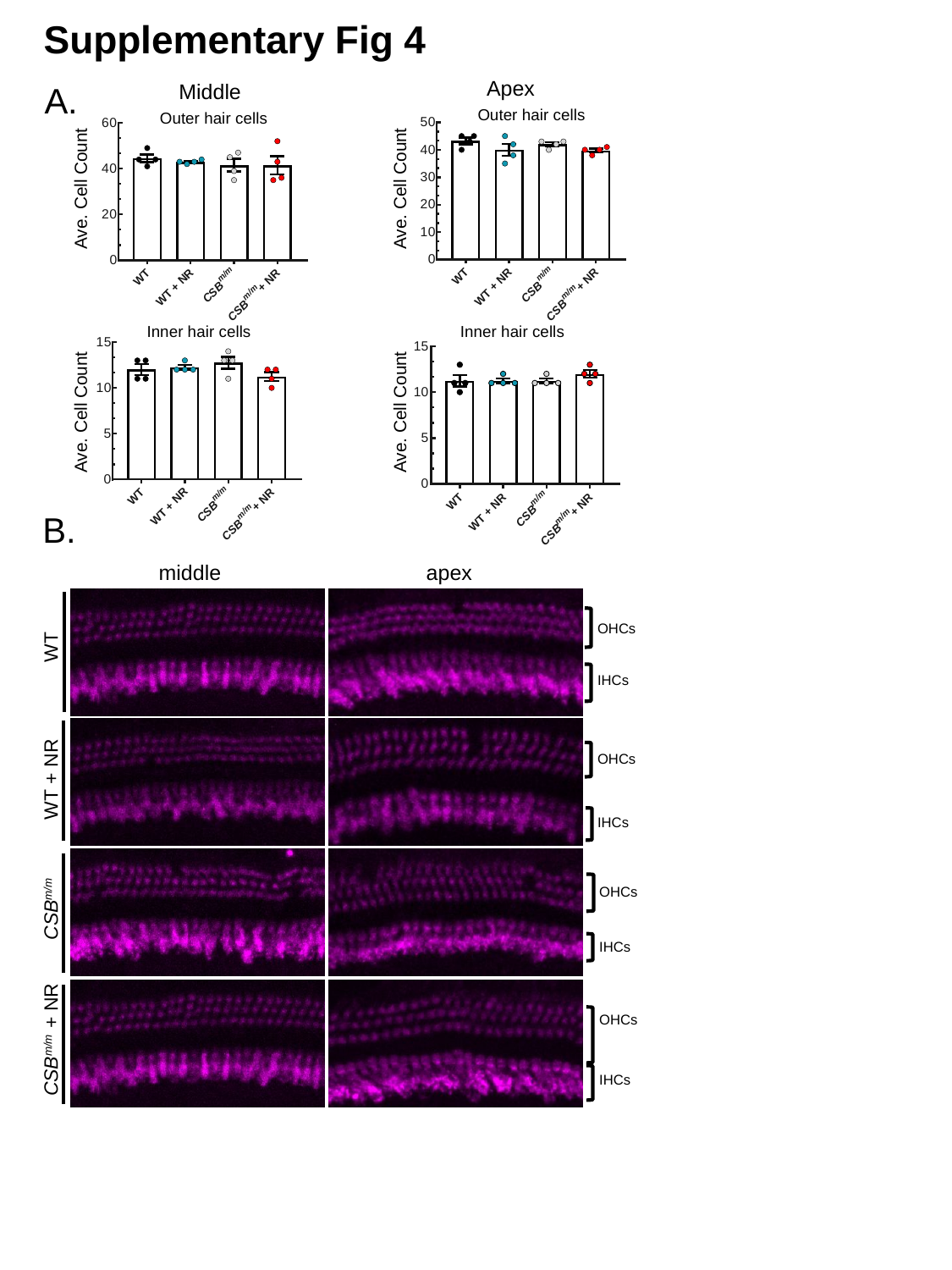

Supplementary Fig 4
A.
Apex
Middle
Outer hair cells
Outer hair cells
Ave. Cell Count
Ave. Cell Count
Inner hair cells
Inner hair cells
Ave. Cell Count
Ave. Cell Count
B.
middle
apex
OHCs
WT
IHCs
OHCs
WT + NR
IHCs
OHCs
CSBm/m
IHCs
OHCs
CSBm/m + NR
IHCs

## Slide 7
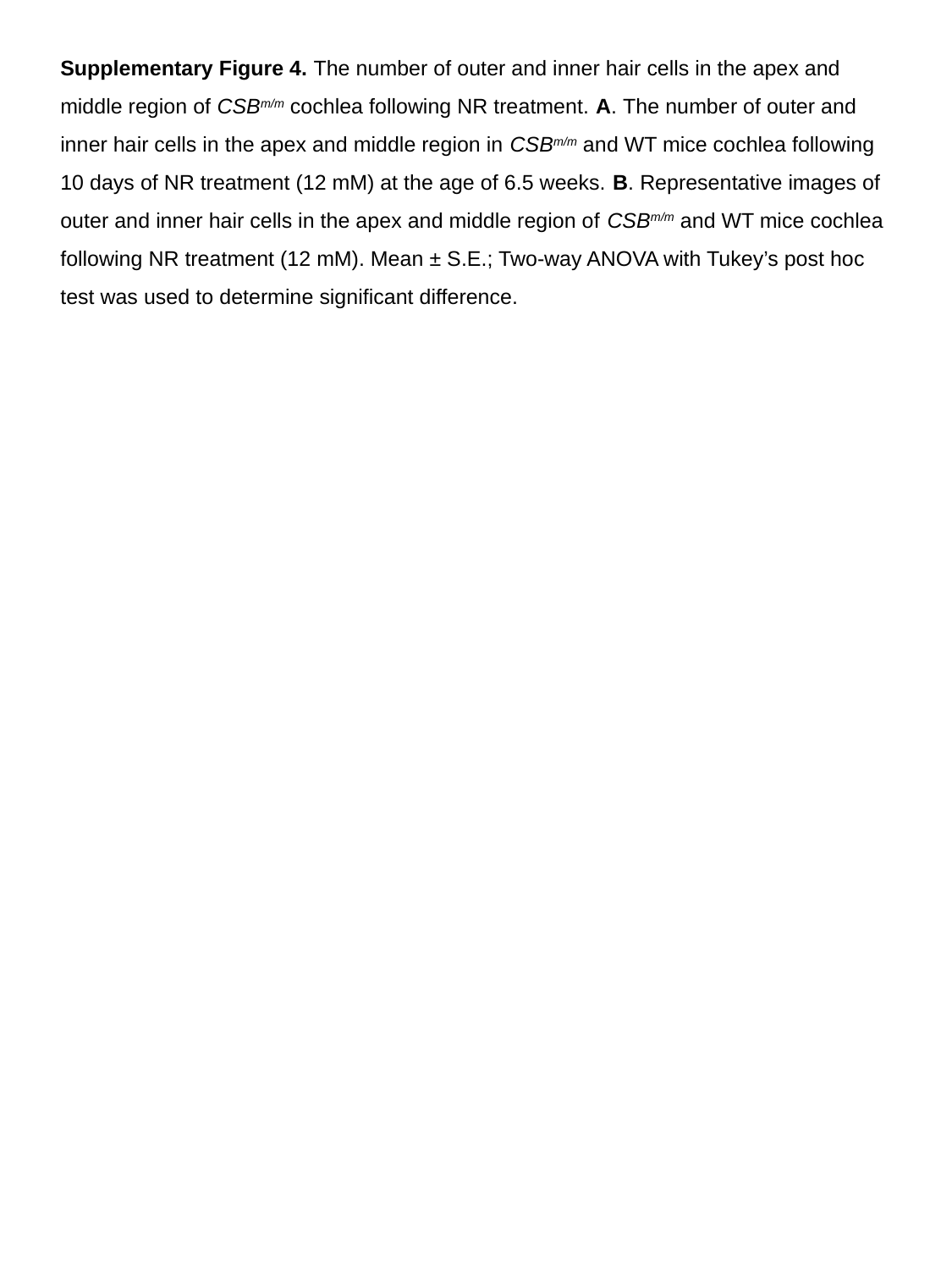

Supplementary Figure 4. The number of outer and inner hair cells in the apex and middle region of CSBm/m cochlea following NR treatment. A. The number of outer and inner hair cells in the apex and middle region in CSBm/m and WT mice cochlea following 10 days of NR treatment (12 mM) at the age of 6.5 weeks. B. Representative images of outer and inner hair cells in the apex and middle region of CSBm/m and WT mice cochlea following NR treatment (12 mM). Mean ± S.E.; Two-way ANOVA with Tukey’s post hoc test was used to determine significant difference.

## Slide 8
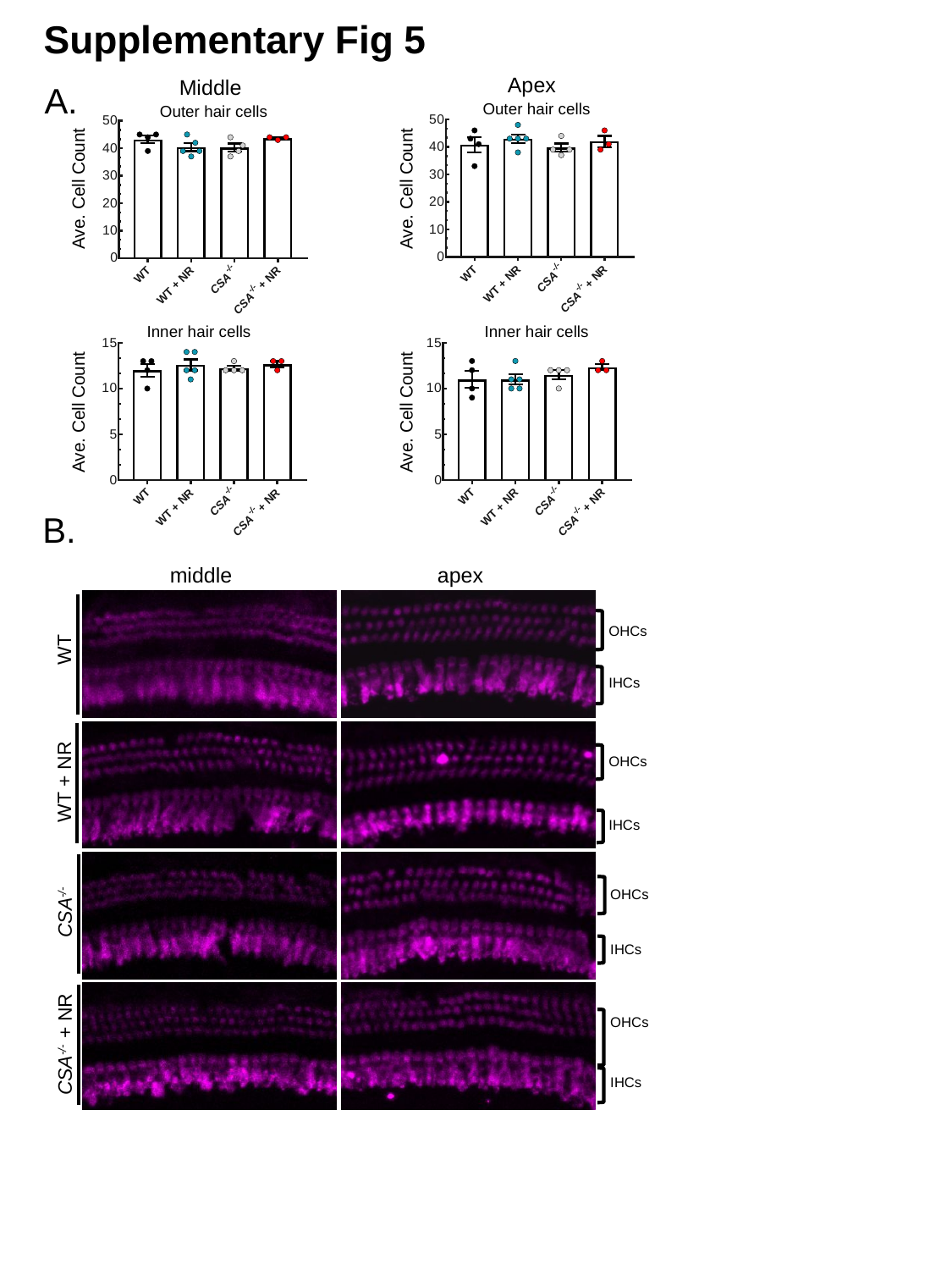

P=0.02
Supplementary Fig 5
A.
Apex
Middle
Outer hair cells
Outer hair cells
Ave. Cell Count
Ave. Cell Count
Inner hair cells
Inner hair cells
Ave. Cell Count
Ave. Cell Count
B.
middle
apex
OHCs
WT
IHCs
OHCs
WT + NR
IHCs
OHCs
CSA-/-
IHCs
OHCs
CSA-/- + NR
IHCs

## Slide 9
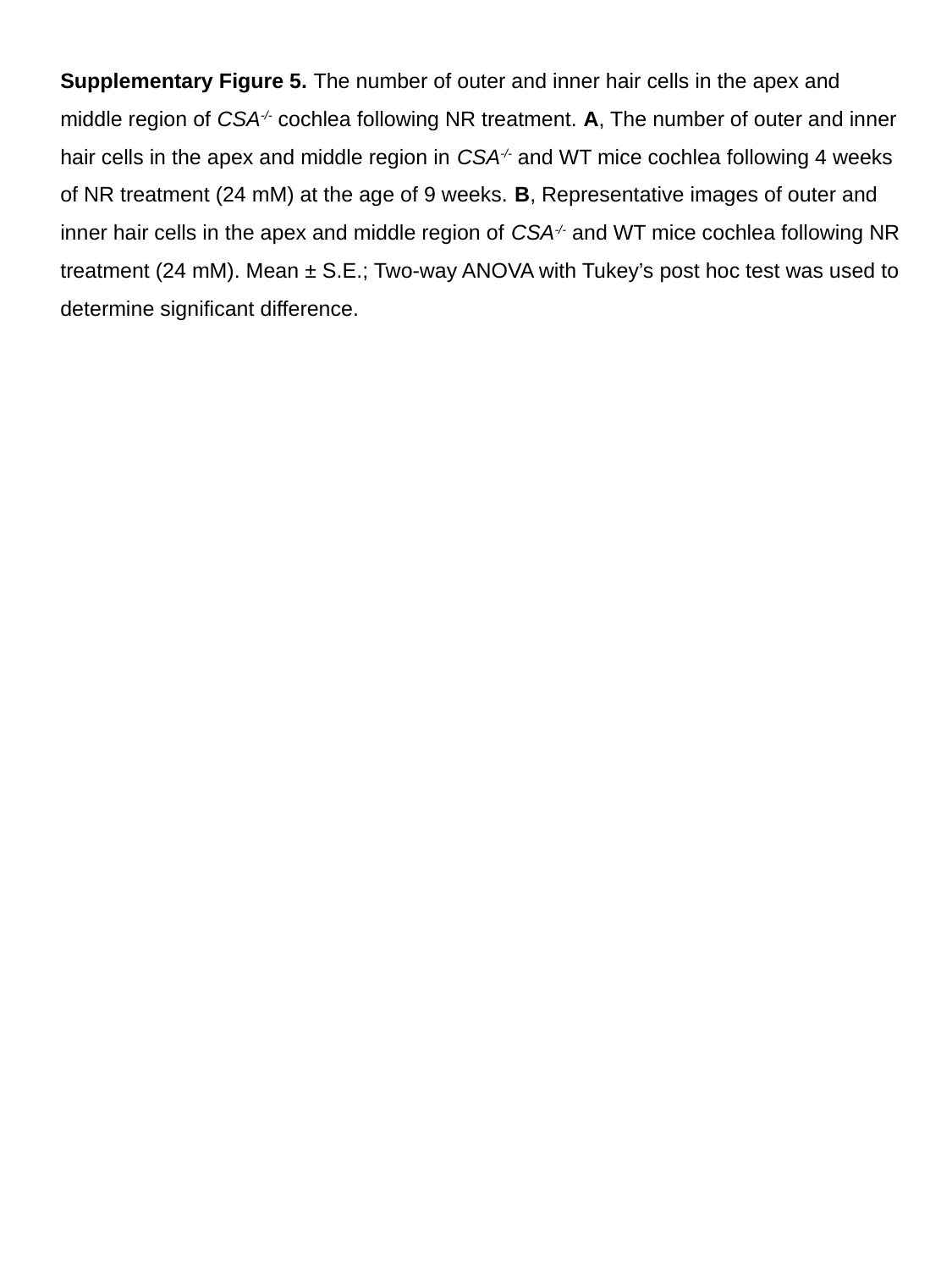

Supplementary Figure 5. The number of outer and inner hair cells in the apex and middle region of CSA-/- cochlea following NR treatment. A, The number of outer and inner hair cells in the apex and middle region in CSA-/- and WT mice cochlea following 4 weeks of NR treatment (24 mM) at the age of 9 weeks. B, Representative images of outer and inner hair cells in the apex and middle region of CSA-/- and WT mice cochlea following NR treatment (24 mM). Mean ± S.E.; Two-way ANOVA with Tukey’s post hoc test was used to determine significant difference.

## Slide 10
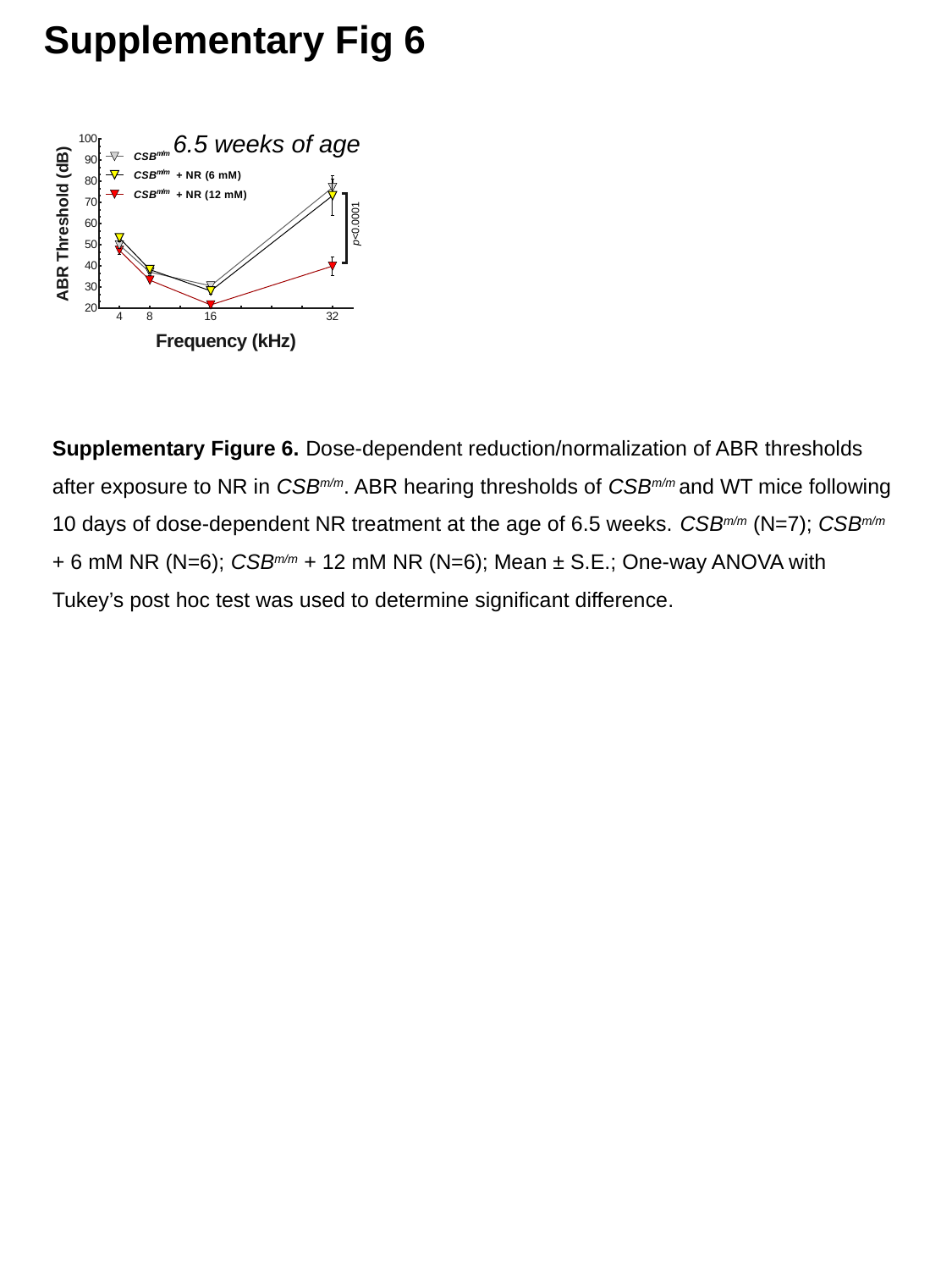

Supplementary Fig 6
6.5 weeks of age
p<0.0001
Supplementary Figure 6. Dose-dependent reduction/normalization of ABR thresholds after exposure to NR in CSBm/m. ABR hearing thresholds of CSBm/m and WT mice following 10 days of dose-dependent NR treatment at the age of 6.5 weeks. CSBm/m (N=7); CSBm/m + 6 mM NR (N=6); CSBm/m + 12 mM NR (N=6); Mean ± S.E.; One-way ANOVA with Tukey’s post hoc test was used to determine significant difference.
